# Ionic, Directional, and Collective Interaction Regimes in Doped B_12_ Clusters: An IQA Perspective

**DOI:** 10.1021/acs.inorgchem.6c02636

**Published:** 2026-08-17

**Authors:** José Manuel Guevara-Vela, Ángel Martín Pendás, Tomás Rocha-Rinza

**Affiliations:** † School of Engineering and Physical Sciences, 3120 Heriot-Watt University, Edinburgh, Scotland EH14 4AS, U.K.; ‡ Departamento de Química Física y Analítica, 16763 Universidad de Oviedo, Julián Claveria 8, Oviedo 33006, Spain; § Instituto de Química, Universidad Nacional Autónoma de México, Circuito Exterior s/n, Ciudad Universitaria, Delegación Coyoacán, Ciudad de México C.P. 04510, Mexico

## Abstract

Doping can alter boron clusters in strikingly different ways, ranging from weak ionic stabilization to strongly localized and delocalized covalent bonding. Yet, these interaction modes are often discussed on a case-by-case basis, without a systematic comparative framework. Thus, we examined structural distortions, charge redistributions, electron sharing, and ionic and covalent contributions in a series of doped B_12_ clusters MB_12_ (M = Li, K, Be, Ca, Sc, Ti, Fe, Cu, Zn, Al, P) using the QTAIM and IQA methods of wave function analysis. These electron-deficient clusters reflect a balance between the energetic cost of deforming the B_12_ scaffold and the stabilizing interaction established with the heteroatom. Four broad interaction classes can be identified concerning individual M–B contacts: weak ionic contacts (LiB_12_ and KB_12_), strongly classical contacts (BeB_12_, CaB_12_ and AlB_12_), strongly covalent contacts (CuB_12_, ZnB_12_ and PB_12_), and a mixed regime in which both classical and exchange-correlation terms are significant and distributed over many boron atoms (ScB_12_, TiB_12_ and FeB_12_), consistent with a more collective mode of interaction with the boron scaffold. The results provided herein offer a chemically transparent framework for the rational selection of dopants in boron-rich clusters by revealing how different elements promote distinct structural motifs and bonding regimes.

## Graphical abstract

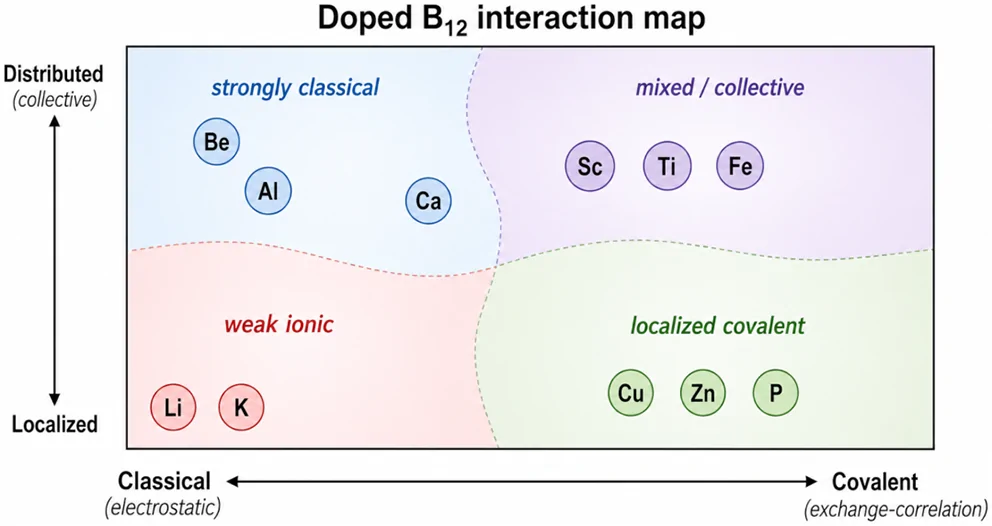

## 1 Introduction

Boron clusters occupy a singular position in cluster chemistry because their structures are not governed by simple two-center bonding patterns, but instead by a delicate interplay of electron deficiency, multicenter bonding, and extensive electronic delocalization.^1,2^ As a result, even relatively small boron aggregates can sustain a remarkable variety of structural motifs and bonding schemes, ranging from quasi-planar and tubular species to cages and fluxional frameworks, often stabilized by combined σ and π aromaticity rather than by conventional localized bonds.^1,3^ This unusual bonding flexibility makes boron clusters exceptionally sensitive to chemical perturbations. Indeed, small changes in composition can redistribute electron density, alter the balance between localized and collective stabilization, and trigger substantial reorganization of the underlying scaffold.^1,3,4^ In this sense, boron clusters are not merely convenient hosts for foreign atoms, but chemically responsive frameworks whose bonding adapts in subtle and often nonintuitive ways to external modification.^3,5^

Doping provides a powerful way of tuning boron clusters by modulating their preferred geometries, charge distribution, and bonding patterns, and therefore the resulting chemistry of these species is remarkably diverse.^6–17^ Depending on the identity of the foreign atom, doping can alter the electronic structure and geometry of boron clusters in markedly different ways, sometimes leaving the parent framework largely recognizable and in other cases inducing substantial reorganization.^6,8–14^ This versatility has given rise to a broad range of doped boron clusters with markedly different geometries, dimensionalities, and electronic structures, spanning species stabilized by alkaline and alkaline-earth metals, motifs substituted with *p*-block elements, and transition-metal-centered wheels, drums, and tubular frameworks.^18–21^ Despite this richness, much of the discussion remains system-specific: individual dopants are often rationalized in terms specific to a given system, while broader comparisons across chemically distinct dopant families remain relatively uncommon.^5,22^ As a result, it remains difficult to distinguish which aspects of dopant behavior reflect general interaction patterns and which arise from the peculiarities of isolated cases.

This structural diversity has also been established experimentally, especially through photoelectron spectroscopy in combination with quantum-chemical structure assignment. Size-selected studies of bare $B_{12}^{-}$ revealed the unusual stability, planarity, and aromatic character of the B_12_ framework, providing one of the early experimental landmarks in the modern understanding of boron-cluster bonding.^23^ Subsequent work on doped B_12_-based clusters showed that different heteroatoms can lead to markedly different bonding situations, as illustrated by ${\mathrm{AlB}}_{6}^{-}/{\mathrm{AlB}}_{11}^{-}$ , ${\mathrm{AuB}}_{12}^{-}$ , and transition-metal-doped ${\mathrm{CoB}}_{12}^{-}$ and ${\mathrm{RhB}}_{12}^{-}$ clusters.^24–26^ More broadly, experimental and theoretical studies from different groups have shown that metal doping can induce structural transformations, fluxionality, and distinct metal–boron bonding regimes in boron clusters.^4,5^ These studies make clear that metal–boron and heteroatom–boron interactions cannot be regarded as minor perturbations of a passive host framework. Rather, the dopant can control the geometry, electron distribution, and aromatic or multicenter bonding pattern of the cluster. They also provide an important reference point for the present work, whose purpose is not to rediscover individual structural motifs, but to compare different dopants on a common real-space energetic basis.

Beyond their fundamental interest, doped boron clusters have attracted attention as candidate building blocks for several classes of functional materials, in ways that depend sensitively on the nature of the dopant–framework interaction. Metal-doped boron clusters have been studied as platforms for reversible hydrogen storage, with Ti-, Sc-, and Y-doped systems among the most promising candidates, wherein the H_2_ binding is governed by a balance of polarization and orbital hybridization at the dopant site.^27–29^ Boron clusters and rings doped with transition metals have also been proposed as magnetic building blocks, including magnetic superatoms and motifs relevant to spintronic materials, with the magnetic response controlled by the extent and character of metal–boron coupling.^22,30,31^ More recently, doped boron clusters, including cage-like motifs, have been explored as nonlinear optical materials and molecular device components, with the corresponding response closely linked to dopant-induced charge redistribution within the cage.^32,33^ In all of these contexts, whether a dopant donates charge diffusely, anchors the framework through a few strong covalent contacts, or couples collectively to the boron scaffold has direct consequences for performance. Still, such distinctions are precisely what conventional descriptors fail to resolve.

The lack of a comparative framework for the rationalization of doped B clusters also exposes a conceptual limitation in how dopant effects are commonly discussed. Descriptions based solely on net charge transfer, formal oxidation states, or qualitative orbital sketches often capture only part of the interaction, and such descriptors may obscure important differences between systems that appear superficially similar. Two dopants may induce comparable charge redistribution while interacting with the boron scaffold via very different balances of electrostatic attraction, covalent exchange, as well as framework-wide delocalization and deformation. In electron-deficient clusters such distinctions are especially important, because stabilization is not necessarily concentrated in a single localized bond, but it may instead be distributed across the network in a more collective manner. A comparative interaction analysis is therefore needed if one wishes to move beyond case-by-case interpretation and to identify chemically meaningful patterns across dopant classes in B clusters. In this context, the Quantum Theory of Atoms in Molecules (QTAIM) and the Interacting Quantum Atoms (IQA) formalisms provide a particularly useful combination. QTAIM offers a rigorous topological description of the electron density, allowing the identification of bonding patterns,^34,35^ whereas IQA builds on the QTAIM partitioning of real space to decompose interatomic stabilization into well-defined, easily interpretable energetic contributions.^36–38^ Taken together, these approaches make it possible to relate definite indicators to the nature of chemical bonding to the underlying balance of electrostatic and exchange-covalent interactions. Thereby, QTAIM and IQA provide a natural framework for distinguishing predominantly ionic, directional covalent, and multicenter, framework-delocalized modes of interaction which involve many boron atoms.

Given these circumstances, we studied a series of doped boron clusters, MB_12_ (M = Li, K, Be, Ca, Al, P, Sc, Ti, Fe, Cu, Zn), chosen to span electropositive *s*-block dopants, more directionally interacting *p*-block elements, and transition metals with distinct patterns of *d*-orbital participation. By keeping the boron host fixed and varying only the identity of the dopant, we seek (i) to isolate how different elements perturb the same electron-deficient framework of boron atoms and (ii) to determine whether their effects can be organized into chemically interpretable interaction regimes. To this end, we combined electronic structure calculations along with QTAIM and IQA analyses of the dopant–framework interactions, focusing on the relative roles of electrostatic stabilization, exchange-covalent contributions, and the extent to which the boron scaffold preserves or reorganizes its collective bonding pattern. In this respect, the QTAIM has been successfully applied to study the chemical bonding scenario of boranes, first in diborane and pentaborane^39^ and then in *closo*-, *nido*-, *arachno*-boranes and *closo*-carboranes.^40^ In particular, ref 40 uses QTAIM to determine charge transfer in these systems, bond orders, sites of nucleophilic and electrophilic attacks along with electron delocalization as a source of stabilization for these compounds. Indeed, electron delocalization and aromaticity are two phenomena which should be taken into account for a proper description of the chemical bonding scenario of the systems addressed herein. Another factor to be taken into consideration for the same purpose, are the different type of metal-B interactions which include electrostatic and charge transfer interactions, as well as dispersion, polarization, localized and delocalized covalent bonding.^3^ Thus, we aim in this paper to provide a compact framework to understand how different dopants reshape the bonding and structure of boron-rich clusters, with direct relevance to the rational selection of heteroatoms in boron-based nanoalloys and related nanomaterials.

## 2 Theoretical Framework

Within the QTAIM, the molecular space is partitioned into atomic basins Ω_*A*_ delimited by zero-flux surfaces in the gradient field of the electron density, ρ­(**r**).^34^ This real-space partition allows the definition of chemically meaningful atomic properties such as charges and electron-sharing indices on a common, rigorous footing. In the present work, the QTAIM atomic charge of atom *A* is defined as

1 $$q\left(A\right)=Z_{A}-\int_{\Omega_{A}}\rho \left(r\right)dr$$

where *Z*_*A*_ is the nuclear charge and the integral gives the number of electrons contained in the basin of atom *A*. Electron sharing between two atoms *A* and *B* is characterized through the delocalization index (DI), which measures the number of pairs of electrons shared between the corresponding atomic basins,

2 $$\mathrm{DI}\left(A,B\right)=\left|2\int_{\Omega_{A}}\int_{\Omega_{B}}\rho_{\mathrm{xc}}\left(r_{1},r_{2}\right)dr_{1}dr_{2}\right|$$

where ρ_xc_(**r**_1_, **r**_2_) is the exchange-correlation density. In this way, QTAIM provides a direct real-space description of charge redistribution, bonding topology, and covalent electron sharing across the MB_12_ series.

The IQA partition of the electronic energy^36–38^ builds upon the QTAIM division of the 3D space in atomic basins and it provides an energy decomposition in real space. In IQA, it is useful to express the formation energy of a given doped cluster as the sum of a deformation (or preparation) term and an interaction term,

3 $$E_{\mathrm{form}}=E_{\mathrm{def}}+E_{\mathrm{int}}$$

where *E*_def_ accounts for the energy required to prepare the boron framework and the heteroatom in the present context for bonding, while *E*_int_ contains the stabilization gained when the prepared fragments interact. Within IQA, this interaction energy can in turn be separated into classical and exchange-correlation contributions,

4 $$E_{\mathrm{int}}=V_{\mathrm{cl}}+V_{\mathrm{xc}}$$

where *V*_cl_ collects the classical electrostatic terms related with ionicity and *V*_xc_ accounts for the exchange-correlation contribution associated with covalent stabilization. This decomposition is particularly useful because it allows us to distinguish whether dopant–framework stabilization arises predominantly from ionic, localized covalent, or more distributed collective modes of interaction.

## 3 Computational Details

The structures of neutral XB_12_ clusters (X = Li, K, Be, Ca, Al, P, Sc, Ti, Fe, Cu, Zn) were explored using a two-step protocol combining global structure searches with DFT refinements. Whenever available, previously reported low-energy motifs from the literature^6–14^ were considered as starting points in order to perform an unbiased exploration of the corresponding potential energy surfaces. Initial candidate geometries were generated with the Cluster code,^41^ which implements a genetic algorithm based on the scheme introduced by Alexandrova and coworkers.^42^ In this approach, an initial population of candidate structures is generated at random, subject to the condition that interatomic distances do not fall below a predefined minimum threshold, and each structure is then locally optimized. The population is subsequently updated iteratively by means of mating operations between low-energy parent structures. Offspring are produced by combining fragments from two randomly rotated parents, after which each offspring is locally optimized. Parent selection is energy-biased, so that structures with the lowest energy are more likely to contribute to the next generation. Duplicate structures are identified using the nuclear repulsion energy as the main duplication criterion, and only distinct minima are retained in the evolving population.

For each doped cluster, the first stage of the search was carried out with the PBE/Def2-SVP^43,44^ approximation, starting from an initial population of 10 candidate structures and allowing the population to evolve for 50 generations. All structures lying within 20 kcal/mol of the lowest-energy candidate identified in this first stage were then taken forward to a second, higher-level refinement. In the second stage, these selected candidates were reoptimized with the PBE0/Def2-TZVP^44,45^ methodology and they were also subjected to an additional 10-generation search and the refinement cycle. Harmonic vibrational frequency calculations were carried out for the optimized structures retained during the search and the refinement procedure in order to confirm that they correspond to genuine minima on the potential energy surface. The final energetic ordering discussed in this work corresponds to electronic energies without Zero-Point-Energy (ZPE) corrections. This choice was made to maintain consistency with the IQA analysis, which is formulated as a partition of the electronic energy and therefore does not naturally incorporate ZPE contributions. Representative low-lying isomers obtained from the structure searches are shown in Figure S1 of the Supporting Information, and the corresponding Cartesian coordinates and electronic energies are provided as downloadable .xyz files through the link given therein.

Because several members of the dopant panel can in principle access different electronic states, searches were carried out considering different spin multiplicities whenever chemically plausible. Alternative spin states were examined during the refinement stage, and the final energetic ordering reported for each composition corresponds to the lowest-energy structures among the multiplicities considered. As an additional test of the robustness of the structural assignments made herein, selected low-lying isomers for representative dopants were further examined with PBE0-D4/Def2-TZVP^46^ calculations in order to assess the possible effect of dispersion corrections on the relative stability of competing minima. In the specific case of ScB_12_, the energy difference between two isomers was less than 1 kcal/mol, and DLPNO–CCSD­(T)/Def2-TZVP^47–53^ single-point energy calculations were therefore carried out to assign the correct energetic ordering. These calculations were used only as sensitivity checks and were not employed in the main IQA workflow, since neither the D4 correction nor the DLPNO–CCSD­(T) electronic structure method have been incorporated into the standard IQA energy partition.

All electronic-structure calculations were performed with Orca version 6.^54–57^ Final reported structures were fully optimized without symmetry constraints. Computational efficiency was improved via the resolution-of-the-identity approximation for the two-electron integrals together with the appropriate auxiliary basis sets.^58,59^ QTAIM and IQA analysis were carried out using the AimAll suite^60^ on the final PBE0/Def2-TZVP electron densities in order to decompose dopant–framework interactions into physically unambiguous electrostatic and exchange-covalent contributions. As an internal consistency check of the IQA calculations, the total molecular energies reported in the WFX files were compared with the reconstructed IQA total energies obtained as ∑_*A*_ *E*_IQA_(*A*). The corresponding values are reported in Table S1 of the Supporting Information. The average and maximum absolute differences are 1.68 × 10^–3^ and 2.81 × 10^–3^ a.u., respectively, confirming the numerical consistency of the IQA energy reconstruction for the systems analyzed in this work. Structural and electronic trends across the series were then analyzed in terms of the extent to which the dopant acts as an ionic stabilizer, a localized covalent anchor, or a more diffuse participant in the bonding manifold of the boron scaffold. Molecular structures were visualized with Avogadro.^61^ Multicenter aromaticity indices were computed with ESIpy from PBE0/Def2-TZVP wave functions generated with PySCF.^62–64^ Atomic partitions based on natural atomic orbitals were used, in line with the formalism implemented in the original ESI-3D program.^65^ For each cluster, the Multicenter Index (MCI) was evaluated for the selected peripheral boron circuit.^66^

## 4 Results and Discussion

In this section, we first present the lowest-energy structures obtained for the MB_12_ series and we discuss the main structural trends across the different dopant families. We then analyze the dopant–framework interactions within the QTAIM and IQA formalisms in order to identify the dominant bonding patterns across the examined systems. Finally, we comment on the most informative outliers and mixed cases and we consider relevant implications for dopant behavior in electron-poor clusters.

### 4.1 Lowest-Energy Structures and Structural Trends across the MB_12_ Series

Figure 1 collects the lowest-energy structures obtained for the examined MB_12_ (M = Li, K, Be, Ca, Sc, Ti, Fe, Cu, Zn, Al, P) series. The bare B_12_ cluster adopts a distorted quasi-planar structure of *C*_3*v*_ symmetry,^67^ which serves as the structural reference for the discussion below. Relative to this motif, some dopants leave the boron framework largely unchanged, whereas others induce substantial reorganization, giving rise to two additional motifs: a boat-like arrangement, as in TiB_12_, and a cup-like arrangement, as in CaB_12_. Figure 1 further shows that similar overall compositions can accommodate qualitatively different dopant environments, ranging from internal positions to peripheral binding modes. These structural trends are discussed below for every dopant class.

**Figure 1:**
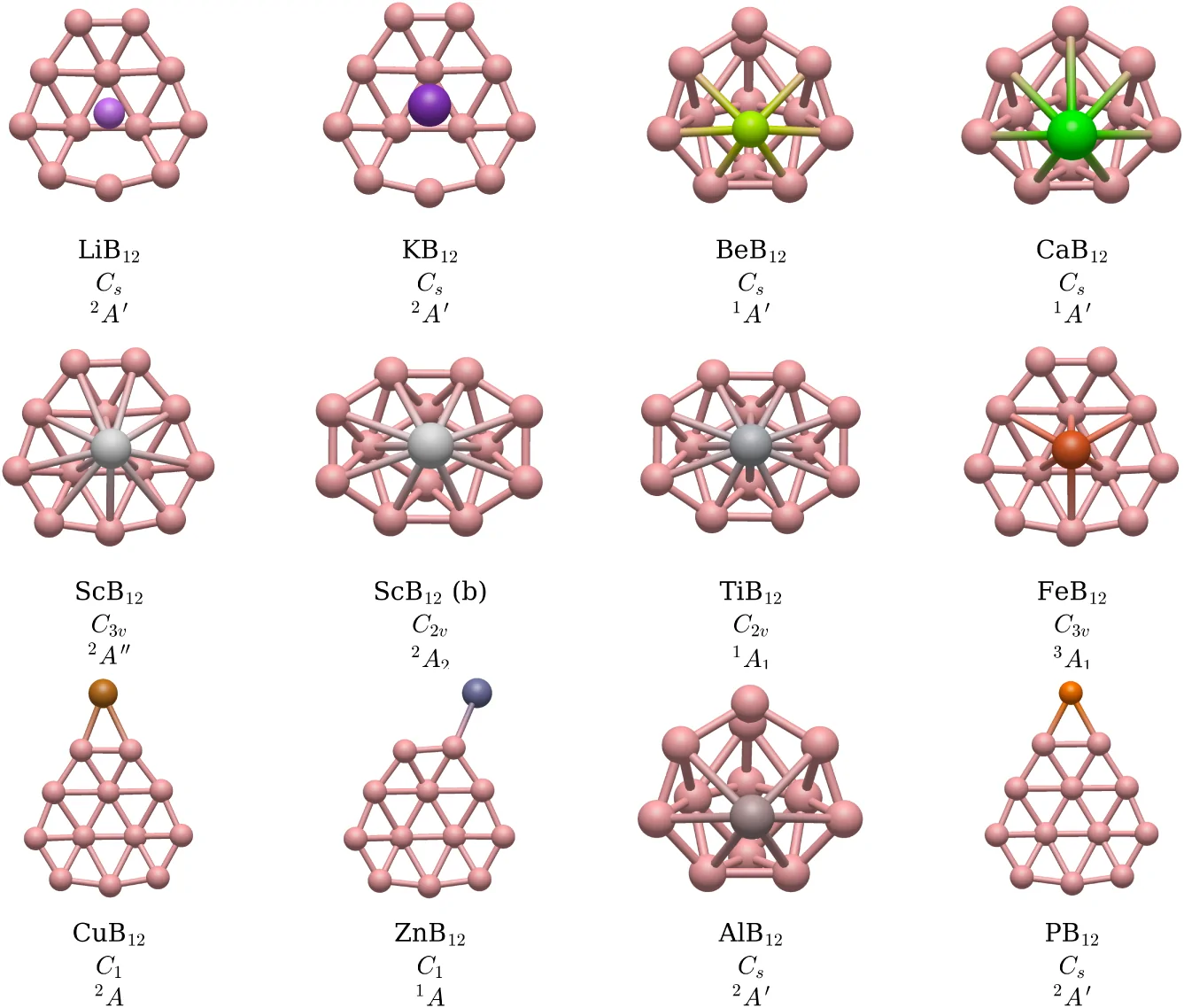
Lowest-lying energy isomers of MB_12_ (M = Li, K, Be, Ca, Sc, Ti, Fe, Cu, Zn, Al, P) clusters computed with the PBE0/Def2-TZVP approximation. The structure of the second lowest energy isomer of ScB_12_ (ScB_12_ (b)) is also included as it sits only 1 kcal/mol above the global minimum. Boron atoms are shown in pink.

Within the electropositive dopant series, LiB_12_, KB_12_, BeB_12_, and CaB_12_ illustrate that closely related elements do not necessarily perturb the boron host in the same way. For LiB_12_ and KB_12_, the lowest-energy structures found here coincide with the minima reported previously in the literature.^8,9^ In both cases, the boron framework largely preserves the structural motif of the bare B_12_ cluster, with the dopant being adjacent to the center of the B_12_ structure. By contrast, BeB_12_ adopts a markedly different arrangement in which the boron scaffold reorganizes into a cup-like motif that allows the Be atom to establish close contacts with six boron atoms. This structure differs from that reported by Feng et al.,^10^ with our lowest-energy structure lying 2 kcal mol^–1^ below the previously proposed minimum. Interestingly, CaB_12_ displays the same lowest-energy structural motif as BeB_12_. To the best of our knowledge, the structure of CaB_12_ has not been previously reported.

We found a different situation for the B clusters doped with transition metals. With the exception of TiB_12_, all of the transition metals preserve the structural motif of the bare B_12_ minimum. TiB_12_ instead adopts a substantially reorganized structure in which the boron framework takes a boat-like arrangement and the Ti atom remains in close contact with all boron atoms. The corresponding Ti–B distances range from 2.1 to 2.5 Å. This structure is consistent with previous reports.^12^ In ScB_12_ and FeB_12_, by contrast, the parent boron motif is retained and the dopant occupies a position in which it is in contact with several B atoms. The structure of FeB_12_ and of ScB_12_, i.e., of half-sandwich, is reminiscent of the complexes CoB_12_^–^ and RhB_12_^–^ which have been previously characterized theoretically and experimentally.^26^ The structure found herein for ScB_12_ is in good agreement with that reported previously by Jin et al.,^7^ whereas an alternative isomer, denoted as ScB_12_(b), lies only 1 kcal/mol above the found global minima and it adopts a reorganized structure closely related to that found for TiB_12_. We compare ScB_12_ and ScB_12_(b) thoroughly later in the paper. To the best of our knowledge, the structure of FeB_12_ has not been reported before. Taken together, TiB_12_, ScB_12_, and FeB_12_ already suggest a mode of metal–framework coupling that is more delocalized than in the edge-bound cases, i.e., Cu, Zn, and P, discussed below. We will establish later the collective mode of interaction of Ti, Sc, and Fe from IQA analyses.^68,69^ CuB_12_ and ZnB_12_ also preserve the B_12_ framework, but unlike ScB_12_ and FeB_12_, the dopant is not embedded in the cluster interior. Instead, Cu and Zn occupy peripheral positions at the edge of the boron scaffold: Cu establishes contacts with two edge boron atoms, whereas Zn interacts with only one. These structures are in agreement with previous reports for CuB_12_ and ZnB_12_.^13,14^

Finally, the *p*-block dopants provide two contrasting examples. AlB_12_ adopts a motif closely related to those found for BeB_12_ and CaB_12_, in which the boron scaffold reorganizes substantially around the dopant, i.e., the B framework adopts a cup-like arrangement. PB_12_, by contrast, retains the structural motif of the bare B_12_ cluster, with the P atom connected only to the edge of the boron framework. Both structures are in agreement with previous reports.^6,11^

Taken together, the structures in Figure 1 show that dopant incorporation into a common B_12_ host can lead either to preservation of the parent motif or to substantial reorganization, depending strongly on the identity of the foreign atom. The energetic differences associated with this structural reorganization vary substantially across the series and will be discussed in more detail below. This observation indicates that the preference to preserve or reshape the boron scaffold by virtue of its interaction with the dopant depends on the balance between the intrinsic stability of the host framework and the interaction established with the dopant. This result is already evident within the *s*-block series, where the optimized minima range from only a mild perturbation of the boron framework in LiB_12_ and KB_12_ to a clear reorganization in BeB_12_ and CaB_12_. The transition-metal-doped clusters display an even broader structural spread, spanning internal, peripheral, and strongly reorganized arrangements within the same dopant family.

In this respect, Popov et al.^26^ wrote *“On the basis of these ideas, one would expect to find similar clusters with a transition metal atom coordinated in the same manner with respect to a quasiplanar boron framework. The boron cluster should maintain a similar planar geometry to that of the undoped boron cluster of the same stoichiometry.”* Nevertheless, our results reveal that there are dopants such as Be, Ti, Ca and Al whose interaction with the B_12_ cluster drive a substantial reorganization of the boron scaffold. These structural differences differences point out that the dopant–framework interactions are not governed by a single bonding pattern and therefore these dissimilarities motivate the real-space analysis presented in the next sections, where QTAIM and IQA are used to examine how these interactions change across the investigated series.

### 4.2 QTAIM Analysis of Dopant–Framework Interactions

The Quantum Theory of Atoms in Molecules (QTAIM) provides a rigorous real-space framework for analyzing chemical bonding through the topological properties of the electron density.^34^ In the present section, we rely on the QTAIM atomic charges and delocalization indices (see eqs 1 and 2), together with the identification of M–B bond critical points (BCPs), to examine charge redistribution, electron sharing, and bonding topology across the MB_12_ series on a common and internally consistent footing.

Table 1 summarizes four QTAIM descriptors for the dopant atom across the MB_12_ series, namely its atomic charge, the largest M–B DI, the sum of all M–B DIs, the standard deviation of the individual M–B delocalization indices, and the number of M–B BCPs. We begin by considering the atomic charges, which provide a first measure of charge redistribution upon doping. Clear trends emerge across the series. Li and K carry large positive charges close to 0.9 au, whereas Be, Ca, Sc, Ti, and Al are even more positively charged, with values between about 1.4 and 1.6 au. Fe is slightly less charged than the alkaline metal cations, while Cu and Zn display only small positive charges. These charge transfers are in good agreement with previous assignations of oxidation states in MB_12_^–^ complexes. The small QTAIM charge of Cu and Zn in the CuB_12_ and ZnB_12_ systems respectively suggest a oxidation state of M^0^, which has been previously assignated to Co and Rh in the complexes CoB_12_^–^ and RhB_12_^–^:^26^ *“the current bonding analyses suggest that the metal atom has a rare oxidation state of M* ^*0*^ *(d* ^*9*^ *) in the MB*_*12*_^*–*^ *complexes, even though six electrons participate in the formation of M–B bonds.”* wherein M = Rh and Co. Still, we would expect slightly larger positive charges for (a) Cu and Zn in CuB_12_ and ZnB_12_ than for (b) Rh and Co in RhB_12_^–^ and CoB_12_^–^ on two grounds (i) the electronegativity of Rh and Co are marginally larger than those of Cu and Zn and (ii) B_12_ should be a more electronegative species than B_12_^–^ due to its aromaticity and charge state. Likewise, P is essentially neutral. We recall at this point that B is an electron deficient atom and therefore, one can expect that a metallic dopant would donate electron density to the B_12_ scaffold. Nevertheless, P is a relatively electronegative atom and such transfer of charge electron density is virtually hindered. We also note that the electronegativity of P and B atoms are 2.19 and 2.03 respectively. In other words, the cluster B_12_ is more electronegative than a single B isolated atom, and its electronegativity is very similar to that of a P atom. These results indicate that the extent of electron transfer from the dopant to the electrically deficient boron framework varies substantially with the identity of the heteroatom. At the same time, simple charge-transfer arguments are not sufficient to rationalize the full range of structural behavior observed in Figure 1. For instance, Li, Sc, and Al all carry large positive charges, yet their dopant-framework interactions differ substantially, as evidenced by the structure of the B framework in its interaction with these metals. On the other hand, the nearly neutral character of P sharply distinguishes this element from the rest of the series. Hence, the charge analysis must be complemented by descriptors that probe electron sharing and bonding topology more directly.

**Table 1:** Selected QTAIM Descriptors Related to the Dopant Atom in the MB_12_ Series Examined Herein^a^

Dopant	*q*(M)	Max. DI(M, B)	∑_B_ DI(M, B)	σ[DI(M, B)]	No. of M–B BCPs
Li	0.90	0.03	0.25	0.01	2
K	0.89	0.05	0.38	0.01	4
Be	1.63	0.12	0.73	0.05	3
Ca	1.49	0.19	1.24	0.06	3
Sc	1.53	0.24	2.56	0.03	1
Ti	1.43	0.46	4.08	0.10	4
Fe	0.76	0.37	3.85	0.05	1
Cu	0.29	0.77	1.80	0.26	1
Zn	0.26	0.61	1.11	0.17	1
Al	1.62	0.24	1.43	0.08	3
P	–0.01	1.19	2.87	0.44	2

a The reported quantities include the QTAIM atomic charge of the dopant, the largest delocalization index between the dopant and any boron atom, the sum of all delocalization indices between the dopant and the boron framework, the standard deviation of the individual M–B delocalization indices, and the number of M–B bond critical points (BCPs). These descriptors provide a compact summary of charge redistribution and local bonding patterns across the series investigated herein.

The remaining descriptors collected in Table 1, namely the largest M–B delocalization index, the summed M–B delocalization index, and the standard deviation of the individual M–B delocalization indices provide a more direct view of how the dopant engages in chemical bonding with the boron framework. Li and K exhibit both small maximum and summed delocalization indices, Max. DI­(M, B) and ∑_B_ DI­(M, B), consistent with weak electron sharing between the dopant and the host. Be, Ca, and Al show larger values for Max. DI­(M, B) and ∑_B_ DI­(M, B) than the alkaline metals, indicating a more substantial covalent character in the dopant–framework interaction, although still well below the most strongly covalently bonded systems. The transition-metal-doped clusters span a broader range of delocalization indices. Sc, Ti, and Fe all display large summed delocalization indices, revealing extensive overall electron sharing with the boron skeleton. Once again, the chemical bonding scenario of these complexes is similar to that of CoB_12_^–^ and RhB_12_^–^ ^26^ for which the authors of ref 26 wrote *“All the π bonds in the B_12_moiety are used to form bonds with the metal atom.”* On the other hand, Cu and Zn are distinguished by comparatively large maximum delocalization indices but more modest summed values and a large standard deviation of DIs, consistent with more localized interactions at the cluster periphery. PB_12_ is especially notable in this respect, since it combines the largest maximum delocalization index of the series with the third largest summed delocalization index, in line with a strong and directional interaction with two boron atoms at the edge of the scaffold. At the same time, the standard deviations of DIS reveal a more complex picture than either descriptor alone. ScB_12_ and FeB_12_, for instance, show large summed delocalization indices despite displaying only one M–B bond critical point, whereas TiB_12_ combines a similarly high summed delocalization index with multiple M–B bond paths. Conversely, KB_12_ present four BCPs between the metallic dopant and the B_12_ cluster, yet, apart from NaB_12_, KB_12_ has the smallest number of delocalized number of electrons and the smallest exchange-correlation contribution $V_{\mathrm{xc}}^{M\cdots B_{12}}$ (top of Figure 4 *vide infra*) between the metallic atom and the B_12_ scaffold. We think that the number of bond critical points is not a good parameter to assess the relevance of the covalent contribution between the metal atom and the B_12_ cluster. We recall at this point that bond critical points might not appear even when there are certainly chemical interactions within electronic systems, e.g., hydrogen bonds in 1,*n*-alkanediols,^70^ a circumstance which warns the use of the number of BCPs in the assessment of the strength (in particular the covalent component) of a given chemical interaction.

These trends are illustrated more explicitly in Figure 2, which shows the summed M–B delocalization indices as a bar plot together with the distributions of the individual M–B delocalization indices as box plots. The left panel emphasizes the overall extent of electron sharing between the dopant and the boron scaffold, whereas the right panel reveals how that sharing is distributed among the different M–B contacts. Read together, the two panels show that similar summed delocalization indices can arise from rather different local patterns. ScB_12_, TiB_12_, and FeB_12_ combine large summed indices with comparatively even distributions, consistent with an evenly distributed covalent bonding network to the boron framework. By contrast, CuB_12_, ZnB_12_, and especially PB_12_ derive a substantial fraction of their total delocalization from a few much stronger contacts, in consistency with a more localized and directional mode of interaction as observed on in the right side of Figure 2 for these clusters. Overall, the delocalization indices point to qualitatively distinct dopant–framework interaction patterns, a point that becomes even clearer in the IQA energy analysis discussed below.

**Figure 2:**
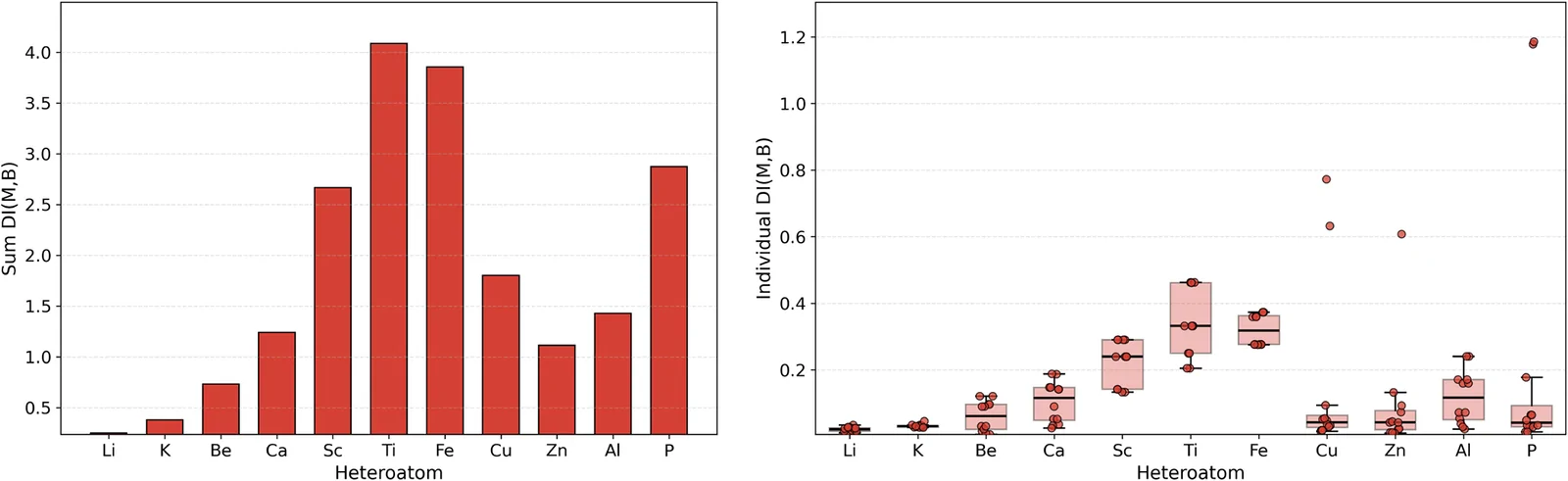
Delocalization indices between the dopant and the boron framework in the MB_12_ series. Left: summed delocalization index between the dopant and the B_12_ skeleton, ∑DI­(M, B). Right: boxplot of the individual M–B delocalization indices.

### 4.3 IQA Energy Decomposition of Dopant–Boron Interactions

In order to gain a more direct view of the interactions between the dopant and the boron framework, we now turn to the IQA formalism. The IQA energy partition has been applied successfully to the analysis of a wide variety of chemical problems, including hydrogen bonding,^71–73^ noncovalent interactions,^74–76^ reaction mechanisms,^77,78^ and the bonding patterns of metal-containing systems such as bimetallic clusters.^79–81^ Here, we employ the IQA decomposition (see eqs 3 and 4) to separate the formation energy into deformation and interaction terms and, in turn, the interaction energy into its classical and exchange-correlation components. This formalism is particularly useful for the MB_12_ series because the structural and QTAIM descriptors discussed above already suggest that the dopant–framework interactions cannot be understood in terms of charge transfer or electron sharing alone. By decomposing both the overall interaction energy and its distribution over the individual M–B contacts, IQA enables us to distinguish systems in which the dopant interacts with the boron host via a few strong localized contacts from those in which the stabilization is spread more broadly across the boron framework. This distinction can be made regardless of whether the dopant–framework interactions are predominantly covalent or ionic, unlike the QTAIM delocalization indices, which primarily probe the covalent aspect of the interaction.

The left side of Figure 3 summarizes the formation energies of the MB_12_ clusters together with their decomposition into deformation and interaction contributions, according to eq 3. In all cases, the dopant–framework interaction is stabilizing, whereas the deformation term is positive and varies strongly across the series. The latter contribution reflects both the structural reorganization of the boron scaffold and the energetic cost of preparing the electronic state of the heteroatom to interact with the B_12_ cluster. This balance is illustrated clearly by BeB_12_ and CaB_12_. Although both clusters have comparable formation energies, BeB_12_ reaches this balance through much larger interaction and deformation contributions than CaB_12_, which combines a less stabilizing interaction energy with a substantially smaller deformation penalty. The left side of Figure 3 therefore shows that the final formation energies of the investigated clusters reflect a compensation between these two terms rather than a simple ordering of the IQA interaction energy itself.

**Figure 3:**
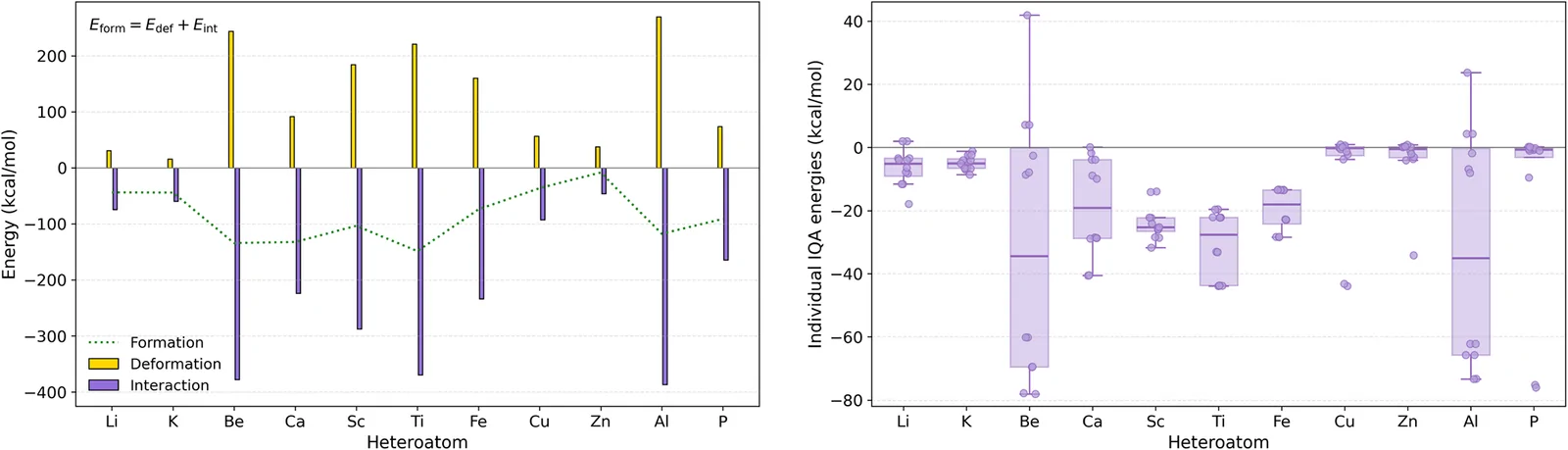
IQA interaction energies between the dopant and the boron framework in the MB_12_ series. Left: total interaction energy between the dopant and the B_12_ skeleton, $E_{\mathrm{int}}^{M-B_{12}}$ along with the accompanying deformation and formation energies. Right: boxplot of the individual M–B interaction energies. The box plots provide a compact summary of their distribution in terms of the median, interquartile range, and overall spread.

The ordering of the IQA interaction energies is itself revealing, since it does not follow either charge transfer or delocalization indices alone. ScB_12_ and FeB_12_, for instance, exhibit very large summed delocalization indices without reaching the most stabilizing dopant–framework IQA interactions, whereas CaB_12_ shows much larger charge transfer but only a moderate IQA interaction energy. The strongest interaction energies are therefore found when charge transfer and the electronic delocalization between the dopant and the B_12_ framework are important, as illustrated by BeB_12_ and AlB_12_. Meanwhile, for example, the interaction of Li and K with B_12_ remains much weaker despite also involving electropositive dopants. Likewise, PB_12_ has a significantly larger IQA interaction energy and therefore a substantially more stabilizing formation energy than BeB_12_ although both adopt edge-bound motifs.

The right side of Figure 3 displays the individual M–B interaction energies for each doped cluster. The points correspond to the explicit pairwise IQA interactions between the dopant and the 12 boron atoms. This figure makes clear that similar total stabilization can arise from very different patterns of chemical bonding. For example, CuB_12_, ZnB_12_, and PB_12_ are dominated by weak contacts with little IQA interaction energy, together with only a small number of much more stabilizing interactions. This circumstance points to a localized mode of binding at the cluster periphery. LiB_12_ and KB_12_ instead exhibit uniformly weak interactions confined to a rather narrow energy window, consistent with a diffuse and weakly structured coupling to the boron framework. BeB_12_ and AlB_12_, by contrast, show much broader distributions, with several strongly stabilizing contacts clearly separated from the rest of the M–B interactions, indicating that a large fraction of the total interaction is concentrated in a limited number of heteroatom–boron contacts. Additionally, we note that two individual M–B interactions are in fact strongly repulsive—one in BeB_12_ and one in AlB_12_—showing that the strongest total stabilization in these systems does not result from uniformly favorable coupling to all boron atoms, but from a markedly anisotropic interaction pattern in which attractive and repulsive contacts coexist. ScB_12_, TiB_12_, and FeB_12_ define a different pattern, in which stabilization comes from a broader set of moderately strong contacts, consistent with a more distributed coupling of the heteroatom to the boron scaffold.

Figure 4 further resolves the dopant–framework interaction by separating the total IQA interaction energy into its classical and exchange-correlation components, both at the level of (i) the full M–B_12_ interaction and (ii) the individual M–B contacts. The top panel reports the total contributions to the IQA interaction energy between the dopant and the boron skeleton, with the classical and exchange-correlation terms stacked for each dopant. This representation shows that the relative balance between both components changes substantially across the series examined herein. In a case by case basis, for about half of the systems the total IQA interaction energy is dominated by the classical term, for which the exchange-correlation contribution is much smaller. ScB_12_ and TiB_12_ exhibit a more even balance between the classical and exchange-correlation components, with both making substantial stabilizing contributions to the total dopant–framework interaction. For FeB_12_, CuB_12_, ZnB_12_, and PB_12_ the stabilization arises almost entirely from the exchange-correlation term. Thus, similar total IQA interaction energies, e.g., Ca···B_12_ and Fe···B_12_, can conceal very different energetic balances, ranging from predominantly classical stabilization to cases in which exchange-correlation effects, i.e., covalency, become dominant.

**Figure 4:**
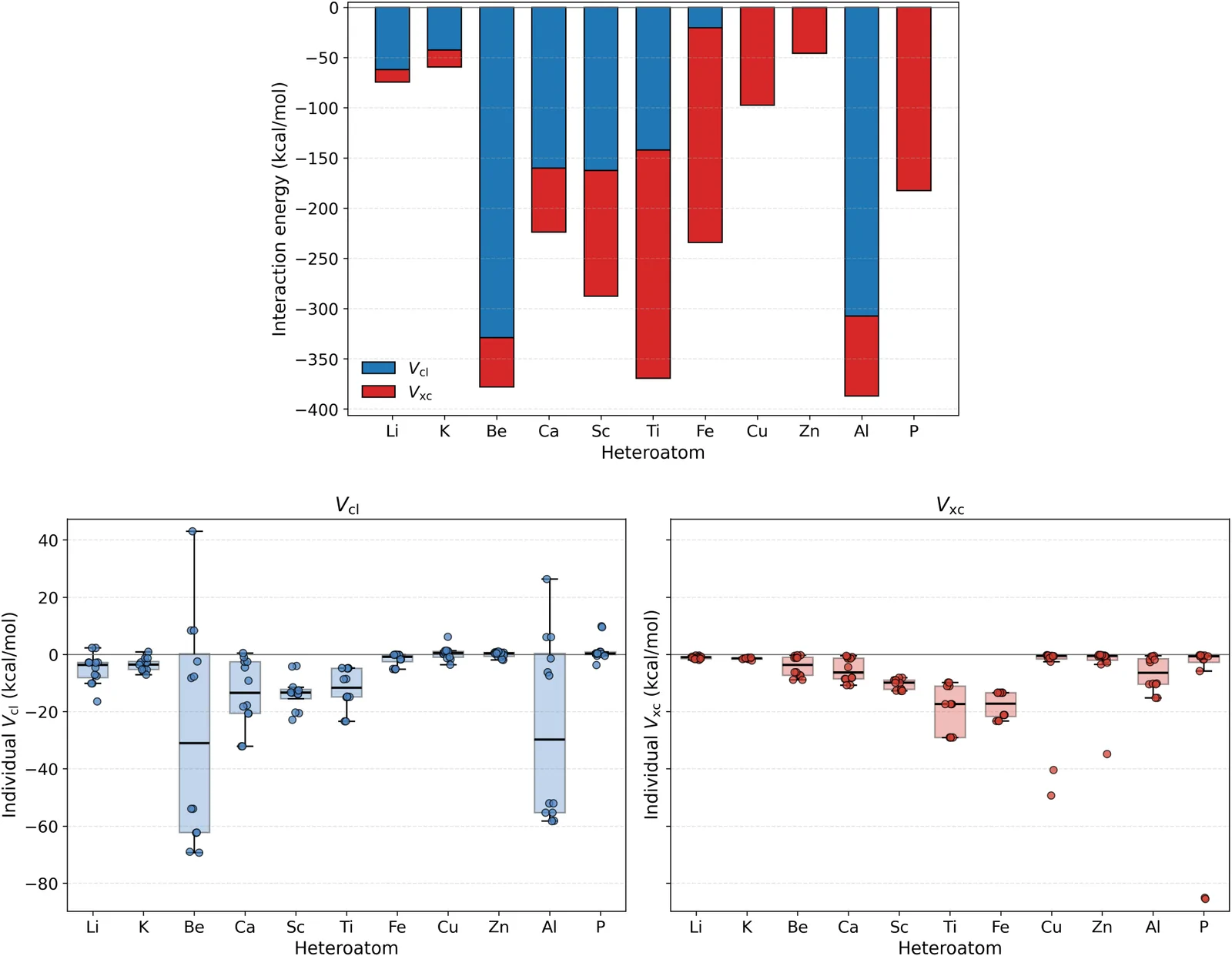
IQA decomposition of the dopant–framework interactions in the MB_12_ series investigated herein. Top: total dopant–framework IQA interaction energy separated into its classical (*V*_cl_, blue) and exchange-correlation (*V*_xc_, red) components. For clarity, positive (repulsive) contributions are omitted from the upper panel. In the lower panels, the points represent the explicit pairwise M–B interactions, whereas the box plots summarize their median, interquartile range, and overall spread. Bottom left: distribution of the individual classical M–B interaction energies. Bottom right: ditto for the exchange-correlation M–B interaction energies.

The two panels in the bottom row of Figure 4 display the corresponding distributions of the individual classical and exchange-correlation components of the M–B contributions for each doped cluster. The classical panel on the left reveals that LiB_12_ and KB_12_ are dominated by many weakly stabilizing contributions. CaB_12_, ScB_12_, and TiB_12_ show a broader set of moderately stabilizing classical contributions, whereas for FeB_12_, CuB_12_, ZnB_12_, and PB_12_ the classical contributions are generally much closer to zero. BeB_12_ and AlB_12_ stand out again, since both clusters display several strongly stabilizing classical contacts, in line with the substantial charge depletion at the dopant atom. One can also notice Be···B_12_ and Al···B_12_ pairs with a very marked repulsive character in BeB_12_ and AlB_12_, which shows that the strong overall stabilization of these clusters is not achieved via uniformly favorable electrostatic interactions. In principle, repulsive IQA classical terms may arise from a variety of sources, including higher-order multipolar effects. Therefore, these classical terms do not need to be reducible to a simple point-charge picture. In the present cases, however, the charge distribution provides already a clear explanation. As shown in Figure 5, both BeB_12_ and AlB_12_ contain a small number of boron atoms with positive QTAIM charges located close to the dopant. The corresponding repulsive contacts therefore arise naturally from the interaction between the positively charged heteroatom and boron sites whose closeness is imposed by the geometry of the cluster. This interpretation is reinforced by comparison with CaB_12_, which adopts the same overall structural motif but it displays neither positively charged boron atoms nor similarly strong repulsive contacts.

**Figure 5:**
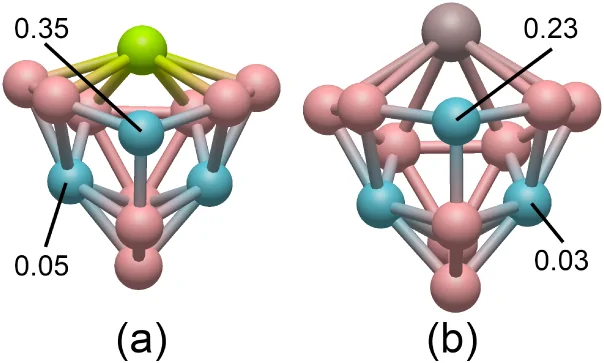
Localization of positively charged boron atoms in (a) BeB_12_ and (b) AlB_12_. Highlighted boron atoms in blue correspond to the QTAIM basins with positive atomic charges (in a.u., shown next to each atom).

The exchange-correlation panel on the bottom right of Figure 4 shows that this component is uniformly small for LiB_12_ and KB_12_, and it remains comparatively modest also in BeB_12_, CaB_12_, and AlB_12_. By contrast, ScB_12_, TiB_12_, and FeB_12_ exhibit many moderately stabilizing exchange-correlation contributions, consistent with a more distributed interaction involving the *d* orbitals of the metal and the boron framework and, in turn, with a more collective mode of interaction with the boron scaffold.^68,69^ We would like to indicate at this point that Figure 1 reports that the B_12_ scaffold in ScB_12_ and FeB_12_ remains virtually unaltered with respect to that of the isolated cluster while the B_12_ structure in the TiB_12_ and AlB_12_ complexes changes substantially to boat- and cup-like configurations respectively. Still, the hapticity of the dopant in the four complexes ScB_12_, FeB_12_, TiB_12_ and AlB_12_ is alike. This observation suggests that the chemical bondings of Sc, Fe, Ti and Al with the B_12_ cluster are of similar nature. Nevertheless, the top part of Figure 1 indicates that whereas the chemical bondings of Sc···B_12_ and Ti···B_12_ are roughly similar with alike contributions from $E_{\mathrm{cl}}^{M\cdots B_{12}}$ and $E_{\mathrm{xc}}^{M\cdots B_{12}}\left(M=\mathrm{Sc},\mathrm{Ti}\right)$ , the chemical bondings Fe···B_12_ and Al···B_12_ are strongly different: the first being mainly covalent while the second largely ionic. These examples illustate that we cannot characterize the chemical bonding within a structure by purely geometrical arguments. CuB_12_, ZnB_12_, and especially PB_12_ display a different pattern, with a small number of markedly stabilizing exchange-correlation contacts together with many much weaker ones, which points to the dominance of a more directional covalent interaction.

### 4.4 Energetic Balance and Motif Selection of B_12_

The structural preferences of the examined doped B_12_ clusters should be interpreted against the experimentally established stability of planar and quasi-planar boron frameworks. In their combined photoelectron spectroscopy and theoretical study of size-selected boron cluster anions, Zhai et al. showed that B_10_–B_15_ clusters are dominated by planar or quasi-planar structures and display aromatic or antiaromatic behavior analogous to that of planar hydrocarbons.^23^ Neutral B_12_ was identified as an unusually stable aromatic cluster with six delocalized π electrons and a large HOMO–LUMO gap, while $B_{12}^{-}$ was found to retain a closely related quasi-planar framework. Although there are boron complexes doped with metals forming molecular wheels such as RhB_9_^–^, IrB_9_^–^, CoB_8_^–^ and RuB_9_^–^ ^82,83^ which have been characterized as aromatic in the basis of photoelectron spectroscopy and density functional theory calculations, there might be departures from the quasi-planarity of B_12_ induced by dopants that entail compensating for a genuine intrinsic preference of the boron scaffold, rooted in aromatic delocalization. This point provides the electronic-structure background for the deformation–interaction balance discussed below.

With this reference in mind, we next compare the relative stability of three B_12_ framework motifs that appear across the doped series: the quasi-planar motif, as in PB_12_; the boat-like motif, as in TiB_12_; and the cup-like motif, as in CaB_12_. Figure 6 shows the relative total electronic energies of these motifs for the neutral, anionic, and dianionic charge states. In agreement with the intrinsic stability of the aromatic B_12_ scaffold discussed above, the quasi-planar structure remains the most stable motif in all three charge states. One might conjecture in this regard, that the aromatic character of B_12_ would make the species B_12_^–^ be nonplanar and unstable. Indeed, the species B_12_^–^ is nonplanar with a nine-membered outer ring and a three-membered inner ring. On the other hand, Romanescu et al.^24^ considered that the nonplanarity of B_12_^–^ might result from *mechanical* effects *“Some non-planarity [of boron clusters] has been traced to antiaromaticity, that is electronic in origin. One aspect that has not been addressed is if the non-planarity can be mechanical in origin, that is, the outer B ring is too small to fit the central atoms.”* To corroborate or disprove this hypothesis, these workers performed an isoelectronic substitution in the B_12_^–^ cluster to form the AlB_11_^–^ cluster whose geometrical configuration, electronic structure and chemical bonding was investigated via photoelectron spectroscopy and quantum chemical calculations. The two most stable structures of AlB_11_^–^ determined via density functional theory are certainly planar and they correspond very well with the experimental observations. The examination of the chemical bonding scenario of one of these isomers revealed that one of them exhibits σ aromaticity while the other is fundamentally an Al atom bonded to the highly aromatic planar cluster B_11_^–^, in the form Al^+^[B_11_]^−^. The authors of ref 24 concluded that *the nonplanarity of B*_*12*_^*–*^ *is mechanical in nature*, videlicet, there are no intrinsic electronic factors which result in the instability of B_12_^–^. Despite the mechanical character of the nonplanarity of B_12_^–^, the energy separation from the boat-like and cup-like arrangements decreases markedly upon reduction of the system. For neutral B_12_, the boat-like and cup-like motifs lie 39.0 and 70.8 kcal mol^–1^ above the quasi-planar minimum, respectively. These gaps decrease to 24.5 and 47.0 kcal mol^–1^ in $B_{12}^{-}$ and to 6.5 and 21.7 kcal mol^–1^ in $B_{12}^{2-}$ .

**Figure 6:**
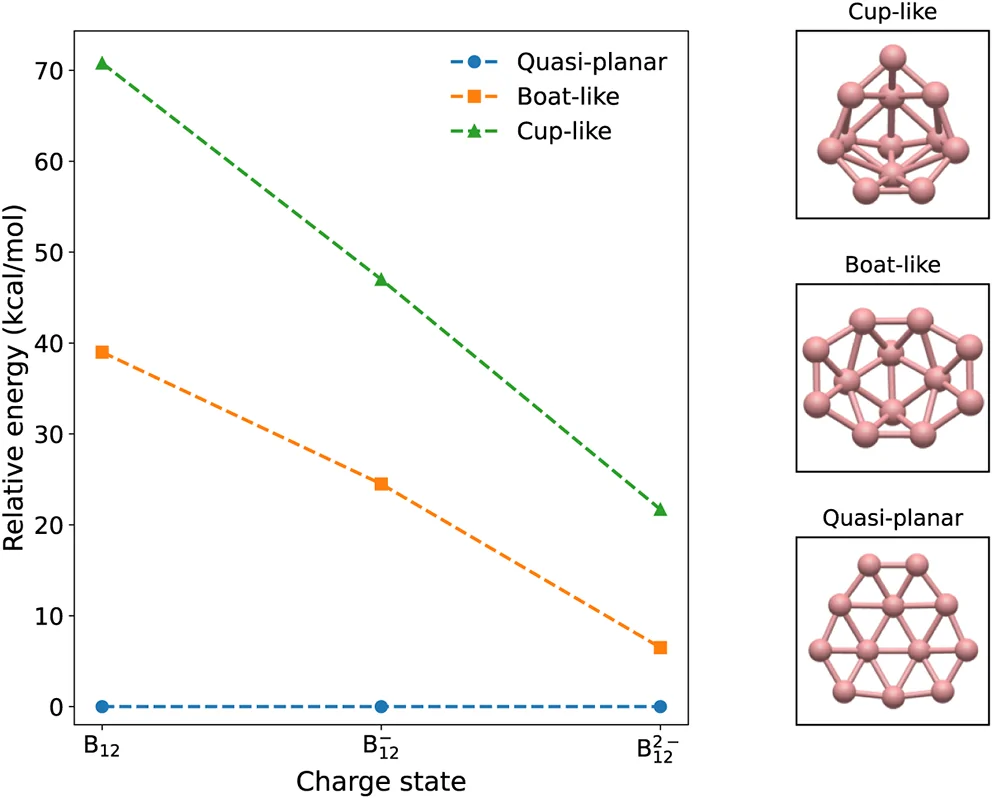
Relative energies of the quasi-planar, boat-like, and cup-like motifs of the B_12_ framework for its neutral, anionic, and dianionic charge states. The corresponding optimized structures of the three reference motifs are shown on the right.

To test whether the extended, quasi-planar boron frameworks exhibit enhanced aromatic character upon doping, we evaluated the MCI for the selected peripheral boron circuit of each complex as reported in Table 2. The complexation with the dopant changes the size of the peripheral boron ring as reported in the third column of Table 2. The clusters containing an external B_9_ ring, which correspond most closely to the quasi-planar motif, show varied (i.e., smaller and larger) values of MCI with respect to the isolated B_12_ cluster. For example, LiB_12_ and KB_12_ display larger B_9_ MCI values than B_12_ because Li and K act mainly as weak ionic stabilizers: they donate electron density to the electron-deficient boron framework while preserving a largely quasi-planar scaffold. In this sense, their effect is qualitatively similar to that observed for B_12_^–^, for which the addition of one electron modestly increases the B_9_ MCI relative to neutral B_12_. ScB_12_ and FeB_12_ also show enhanced B_9_ cyclic delocalization, but for a different reason: these dopants combine charge donation with a more distributed dopant–framework coupling, involving several M–B contacts rather than a single localized bond. In contrast, CuB_12_, ZnB_12_, and PB_12_ do not enhance the B_9_ MCI relative to B_12_ because their interaction with the B_12_ cluster is a localized and directional covalent bond. In these cases, especially for PB_12_, strong covalent anchoring at the edge of the cluster locally stabilizes the dopant–boron contacts but it does not reinforce the cyclic delocalization of the whole B_9_ perimeter. Although the complexes CuB_12_, ZnB_12_, and PB_12_ present the smallest values of MCI in Table 2, the corresponding dopants do not disrupt severely the aromaticity of the B_12_ moiety, in a similar fashion to the complexes AuB_12_^–^ and (BO)­B_12_^–^.^25^ The system ScB_12_(b) presents a reduction of the peripheral ring with respect to ScB_12_. The comparison between ScB_12_ and ScB_12_(b) shows that even the same dopant can give similar aromaticity patterns even when the connectivity of the boron scaffold changes. The eight-membered outer ring of TiB_12_ presents a smaller MCI value than its counterpart in ScB_12_(b). The compact B_7_-ring systems BeB_12_, AlB_12_, and CaB_12_ show the largest MCI values, but they should be viewed as a separate structural class rather than as direct analogues of the quasi-planar B_9_ systems.

**Table 2:** Multicenter Indices (MCI) for Selected Peripheral Boron Rings of B_12_ and MB_12_ Clusters

System	Scaffold class	Ring size	MCI
B_12_	reference B_9_	9	8.20 × 10^–4^
$B_{12}^{-}$	anionic B_9_	9	9.70 × 10^–4^
KB_12_	extended B_9_	9	1.24 × 10^–3^
LiB_12_	extended B_9_	9	1.63 × 10^–3^
FeB_12_	extended B_9_	9	1.12 × 10^–3^
ScB_12_	extended B_9_	9	1.88 × 10^–3^
ZnB_12_	extended B_9_	9	7.30 × 10^–4^
CuB_12_	extended B_9_	9	7.60 × 10^–4^
PB_12_	extended B_9_	9	5.40 × 10^–4^
ScB_12_(b)	compact B_8_	8	1.84 × 10^–3^
TiB_12_	compact B_8_	8	9.10 × 10^–4^
CaB_12_	compact B_7_	7	9.52 × 10^–3^
AlB_12_	compact B_7_	7	1.02 × 10^–2^
BeB_12_	compact B_7_	7	1.20 × 10^–2^

The trend shown in Figure 6 provides a useful reference for understanding the structures of the doped clusters. Charge donation to the boron framework lowers the energetic cost of accessing reorganized motifs, particularly the cup-like and boat-like arrangements, but it does not by itself invert the intrinsic preference for the quasi-planar scaffold. The observed structure of a doped cluster therefore depends on whether the additional dopant–framework stabilization is sufficient to compensate the cost of moving away from the aromatic B_12_ reference motif. This interpretation is consistent with the IQA results discussed above (Figure 4), where the cup-like systems BeB_12_, CaB_12_, and AlB_12_ display some of the largest overall dopant–framework classical interaction energies, whereas more weakly interacting dopants remain associated with quasi-planar motifs.

A particularly clear case is provided by ScB_12_ and ScB_12_(b). These two isomers are nearly isoenergetic, but differ in the geometry of the boron framework: ScB_12_ retains the quasi-planar motif, whereas ScB_12_(b) adopts a boat-like arrangement. Despite lying slightly above the global minimum in total energy, ScB_12_(b) displays a more stabilizing total dopant–framework IQA interaction energy than ScB_12_ by 34.7 kcal mol^–1^, with both the classical and exchange-correlation components becoming more favorable by 18.2 and 16.5 kcal mol^–1^, respectively. Consistent with this, ScB_12_(b) also shows slightly greater charge transfer from the dopant to the boron scaffold than ScB_12_ (1.57 vs 1.52 au).

This comparison shows that reorganization of the boron framework can enhance the interaction with the heteroatom, but that this enhanced interaction is not automatically sufficient to determine the global minimum. In ScB_12_(b), the additional dopant–framework stabilization is offset by the energetic penalty associated with reorganizing the B_12_ scaffold, so that the quasi-planar ScB_12_ structure remains slightly preferred. More generally, Figure 6 and the ScB_12_/ScB_12_(b) comparison show that electron addition does not erase the intrinsic preference for the aromatic quasi-planar framework, but it does make reorganized motifs substantially more accessible. The final structure of the doped cluster is therefore governed by a deformation–interaction balance between the intrinsic stability of the boron scaffold and the stabilizing interaction recovered upon binding the heteroatom.

### 4.5 M–B Interactions Taxonomy

Taken together, the individual M–B interactions can be organized into four broad classes. The first category corresponds to weak contacts, for which both the classical and exchange-correlation contributions remain small although the former contribution remains dominant; LiB_12_ and KB_12_ are the clearest examples of this regime. Many of the individual contacts in CuB_12_, ZnB_12_, and PB_12_ fall into this category. A second class is formed by strongly classical contacts, in which the stabilization is largely governed by the electrostatic component. This pattern is most evident in BeB_12_ and AlB_12_, where several M–B interactions are strongly stabilizing at the classical level. A third class corresponds to strongly covalent contacts, characterized by a large exchange-correlation contribution concentrated in only a few M–B pairs. This situation is clearly seen in CuB_12_, ZnB_12_, and especially in PB_12_. Finally, the early transition-metal systems ScB_12_, TiB_12_, and FeB_12_ define a mixed regime in which classical and exchange-correlation terms may be significant (see the top of Figure 4) and these components distributed over many boron atoms rather than concentrated in only one or two contacts, consistent with a more collective mode of interaction with the boron scaffold.^68,69^ This classification aids in the understanding why similar overall dopant–framework stabilization may arise from very different local interaction patterns, and therefore why structurally distinct motifs can become preferred for different reasons across the MB_12_ series. Importantly, within the IQA framework, classical and exchange-correlation terms should not be viewed as opposite ends of a single scale, since electrostatic stabilization and covalency constitute distinct aspects of the interaction and they do not reinforce or weaken each other in parallel.^38,84^

Across the MB_12_ series, dopant behavior is governed by two distinct but intertwined factors, namely the ability of the foreign atom to donate electronic density to the electron-poor boron framework, and the energetic cost of reorganizing the boron scaffold. Highly electropositive dopants such as Li and K are effective donors, but their interaction with the framework remains largely weak and nondirectional. Be, Ca, and Al also transfer substantial charge, yet Be and Al in particular interact with the B_12_ cluster via the formation of a limited number of very strong contacts. By contrast, Cu, Zn, and especially P show that strong local covalent interactions may arise accompanied by a comparatively small charge transfer. Finally, Sc, Ti, and Fe occupy an intermediate but especially interesting regime, where substantial charge donation is accompanied by broadly distributed covalent coupling to the boron scaffold. For the design of heteroatomic and bimetallic clusters, this distinction is essential whenever one seeks to decide whether a dopant should preserve the underlying boron framework while tuning its electronic structure, or instead act as a structure-directing agent that promotes scaffold reorganization and a different mode of bonding. In this sense, the present IQA-based analysis provides a chemically transparent basis for dopant selection in boron-rich nanoalloys and related bimetallic clusters.

Finally, the results presented herein allow us to conjecture about the direction of the synthesis of the complexes examined in this investigation. The complexes with the largest formation energies are BeB_12_, CaB_12_, AlB_12_ and TiB_12_. The formation of the first three mentioned systems results from strongly interacting systems dominated by electrostatic stabilization whereas the generation of TiB_12_ stems from a collective distributed set of covalent interactions wherein classical electrostatics make a substantial contribution as shown in the top of Figure 4. We also note that BeB_12_, CaB_12_, AlB_12_ are the complexes with the most aromatic rings according to Table 2. The four systems involve a substantial reorganization of the B_12_ scaffold as revealed in Figure 1. Additionally, BeB_12_, AlB_12_ and TiB_12_ are the complexes with the largest deformation energies as observed in the yellow bars in the left of Figure 3. These energy penalties due to the distortion of the B_12_ scaffold are more than compensated by a largely stabilizing $E_{\mathrm{int}}^{M\cdots B_{12}}$ IQA interaction energy. These results indicate that the successful synthesis of MB_12_ might involve a substantial deformation of the B_12_ cluster and they are to be contrasted with those of Popov et al.^26^ wherein complexes of Rh and Co with B_12_^–^ *“possess half-sandwich-type structures with the quasi-planar B*_*12*_ *moiety coordinating to the metal atom.”* Furthermore, the large deformation that we found in the B_12_ cluster within the BeB_12_ complex might also have important consequences for the structure of Be···borophene put forward by Kang and co-workers.^85^ The authors wrote *“It is worth noting that due to planar structure and chemical bonding characteristics of BeB*_*16*_^*–*^ *cluster, also inspired by fascinating prospect of two-dimensional monolayer metallo-borophene, we successfully build a schematic of possibility of metallo-borophene (not optimized) based on BeB*_*16*_^*–*^ *unit cluster presented in* Fig. S6 *of* Supplementary Information, *which indicated the BeB*_*16*_^*–*^ *cluster is a potential motif for metallo-borophene.”* Nevertheless, and as mentioned before, the interaction of Be with B_12_ distorts strongly the B_12_ cluster. This deformation could make the structure of Be···borophene, put forward in ref 85 unfeasible. Indeed, the synthesis of metallo-borophene has proved elusive^86^ and to the best of our knowledge, the only metallo-borophene synthesized until now is CuB_4_.^87^ In this regard, it is our opinion that the synthesis of a metallo-borophene requires the inclusion of a metallic atom able of forming a network of distributed covalent bonds without disrupting substantially the structure of the B_12_ structure. The results presented in this investigation indicate that Fe might be a good candidate for such purpose.

## 5 Conclusions

We have analyzed the chemical bonding patterns in a series of doped B_12_ clusters, MB_12_ (M = Li, K, Be, Ca, Al, P, Sc, Ti, Fe, Cu, Zn), by combining structural analysis with QTAIM descriptors and the IQA energy decomposition. The examination of these compounds presented herein indicated that the chemical bonding scenario of these species cannot be inferred by merely geometrical considerations such as hapticity. Indeed, our results show that neither charge transfer, nor the presence of M–B bond critical points, nor delocalization indices alone are sufficient to describe the full range of dopant–framework interactions in the MB_12_ series examined herein. For example, the IQA partition of the electronic energy revealed unexpected repulsive contacts between the dopants and a few B atoms. Furthermore, the determination of charge transfer between the interacting species pointed out that the B atoms exert cooperative effects concerning electronegativity, i.e., B_12_ is more electronegative that a single isolated atom and it has an electronegativity comparable with that of phosphorus. The examination of charge transfer also gave insights about the oxidation states of the dopants in the examined clusters. Still, the main outcome of this study is a chemically meaningful taxonomy of dopant–framework interactions in boron-rich clusters. At the level of the individual M–B contacts, four broad interaction classes can be identified: weak contacts (LiB_12_ and KB_12_), for which both the classical and exchange-correlation contributions remain small although the former component is dominant; strongly classical contacts (BeB_12_, CaB_12_ and AlB_12_), dominated by electrostatic stabilization; strongly covalent contacts (CuB_12_, ZnB_12_ and especially PB_12_), characterized by large exchange-correlation stabilization concentrated in a few M–B pairs; and a mixed regime (ScB_12_, TiB_12_ and FeB_12_), in which both classical and exchange-correlation terms may be significant and distributed over many boron atoms, consistent with a more collective mode of interaction with the boron scaffold. This classification provides a comparative framework for understanding how different dopants interact with the same boron host and produce element-specific structural and bonding outcomes.

Although experimentally examined boron coordination complexes such as CoB_12_^–^ and RhB_12_^–^ exhibit half-sandwich structures wherein the B_12_ moiety is virtually unaltered, we found that there are dopants which induce a strong structural reorganization and changes in the aromatic character of the B_12_ unit. Moreover, the results presented herein show that the final formation energy of a doped boron cluster emerges from the interplay between the intrinsic energetic preferences of the B_12_ framework and the stabilizing interaction with the heteroatom. This perspective helps to explain why chemically related dopants, such as Li and Be or Sc and Ti, can yield qualitatively different ground-state motifs: relatively small changes in electron donation, bonding strength, or interaction distribution can tip the balance between preserving and reorganizing the boron framework. In the same way, increasing the electronic density of the boron scaffold lowers the intrinsic energetic penalty for accessing more distorted motifs, making reorganized structures progressively more competitive when they can also support stronger dopant–framework interactions.

The chemical message that emerges from the present analysis is that dopants in boron-rich clusters should not be regarded simply as electron reservoirs. Their role depends on how they redistribute charge, anchor locally, or couple to the framework as a whole. The distinctions made in this paper provide concrete design criteria for boron-rich nanoalloys and related bimetallic clusters, particularly when the target property depends on whether the dopant should preserve the boron scaffold, reshape it, or engage it through a broader collective interaction. These considerations might have relevant consequences for the blueprint and synthesis of structurally diverse, two- and three-dimensional boron-based materials with tunable electronic properties.

## Supplementary Material



## Notes

(i) Links to xyz files (TXT) of the local minima populations of the MB_12_ (M = Li, K, Be, Ca, Sc, Ti, Fe, Cu, Zn, Al, P) clusters addressed in this investigation; (ii) assessment of the quality of the recovery of the electronic energy by means of the IQA partition of this quantity; (iii) representative low-lying isomers of the MB_12_ clusters considered in this work, with M = Li, K, Be, Ca, Sc, Ti, Fe, Cu, Zn, Al, and P (PDF)
