# Supplementary material for: Ionic, Directional, and Collective Interaction Regimes in Doped B12 Clusters: An IQA Perspective
Source: Inorg Chem. 2026 Aug 17;65(34):19865–78. doi: 10.1021/acs.inorgchem.6c02636 (PMC13536941; doi:10.1021/acs.inorgchem.6c02636)
Supplement: Supplementary file 1 [file ic6c02636_si_001.pdf]

Ionic, directional, and collective interaction regimes in doped B_{12} clusters: an IQA perspective
Electronic Supplementary Information

José Manuel Guevara-Vela[a], Ángel Martín Pendás[b], Tomás Rocha-Rinza[c,*]

[a] *School of Engineering and Physical Sciences, Heriot-Watt University, Edinburgh EH14 4AS, Scotland, U.K.*
[b] *Departamento de Química Física y Analítica, Universidad de Oviedo, Julián Claveria 8, 33006, Oviedo, Spain.*
[c] *Instituto de Química, Universidad Nacional Autónoma de México, Circuito Exterior s/n, Ciudad Universitaria, Delegación Coyoacán C.P. 04510, Ciudad de México, Mexico.*

1. xyz files

In this link

`https://doi.org/10.5281/zenodo.21035557`

the interested reader can download the xyz files of the local minima populations of the MB_{12} (M = Li, K, Be, Ca, Sc, Ti, Fe, Cu, Zn, Al, P) clusters addressed in this investigation.

*To whom correspondence should be addressed: trocha@iquimica.unam.mx

2. Quality of the recovery of the electronic energy

Table S1: Consistency check between the total molecular energies reported in the WFX files and the reconstructed IQA total energies obtained as $\sum_A E_{\mathrm{IQA}}(A)$. Energies are reported in atomic units.

System	E_{WFX}	$\sum_A E_{\mathrm{IQA}}(A)$	ΔE	$\lvert\Delta E\rvert$
AlB_{12}	-539.978365547	-539.978651880	2.8633×10^{-4}	2.8633×10^{-4}
B_{12}	-297.654275588	-297.653171050	-1.1045×10^{-3}	1.1045×10^{-3}
BeB_{12}	-312.390993763	-312.391408390	4.1463×10^{-4}	4.1463×10^{-4}
CaB_{12}	-975.128313880	-975.127324720	-9.8916×10^{-4}	9.8916×10^{-4}
CuB_{12}	-1937.956251500	-1937.954077900	-2.1736×10^{-3}	2.1736×10^{-3}
FeB_{12}	-1561.207930128	-1561.205287400	-2.6427×10^{-3}	2.6427×10^{-3}
KB_{12}	-897.463522950	-897.462293790	-1.2292×10^{-3}	1.2292×10^{-3}
LiB_{12}	-305.191037451	-305.189013310	-2.0241×10^{-3}	2.0241×10^{-3}
PB_{12}	-638.934555748	-638.932734280	-1.8215×10^{-3}	1.8215×10^{-3}
ScB_{12}	-1058.233167421	-1058.231671900	-1.4955×10^{-3}	1.4955×10^{-3}
TiB_{12}	-1146.974395683	-1146.973245900	-1.1498×10^{-3}	1.1498×10^{-3}
ZnB_{12}	-2076.811921268	-2076.809109200	-2.8121×10^{-3}	2.8121×10^{-3}

The signed difference is defined as $\Delta E = E_{\mathrm{WFX}} - \sum_A E_{\mathrm{IQA}}(A)$. The average and maximum absolute differences are 1.68×10^{-3} and 2.81×10^{-3} a.u., respectively, corresponding to 1.05 and 1.76 kcal mol^{-1}.

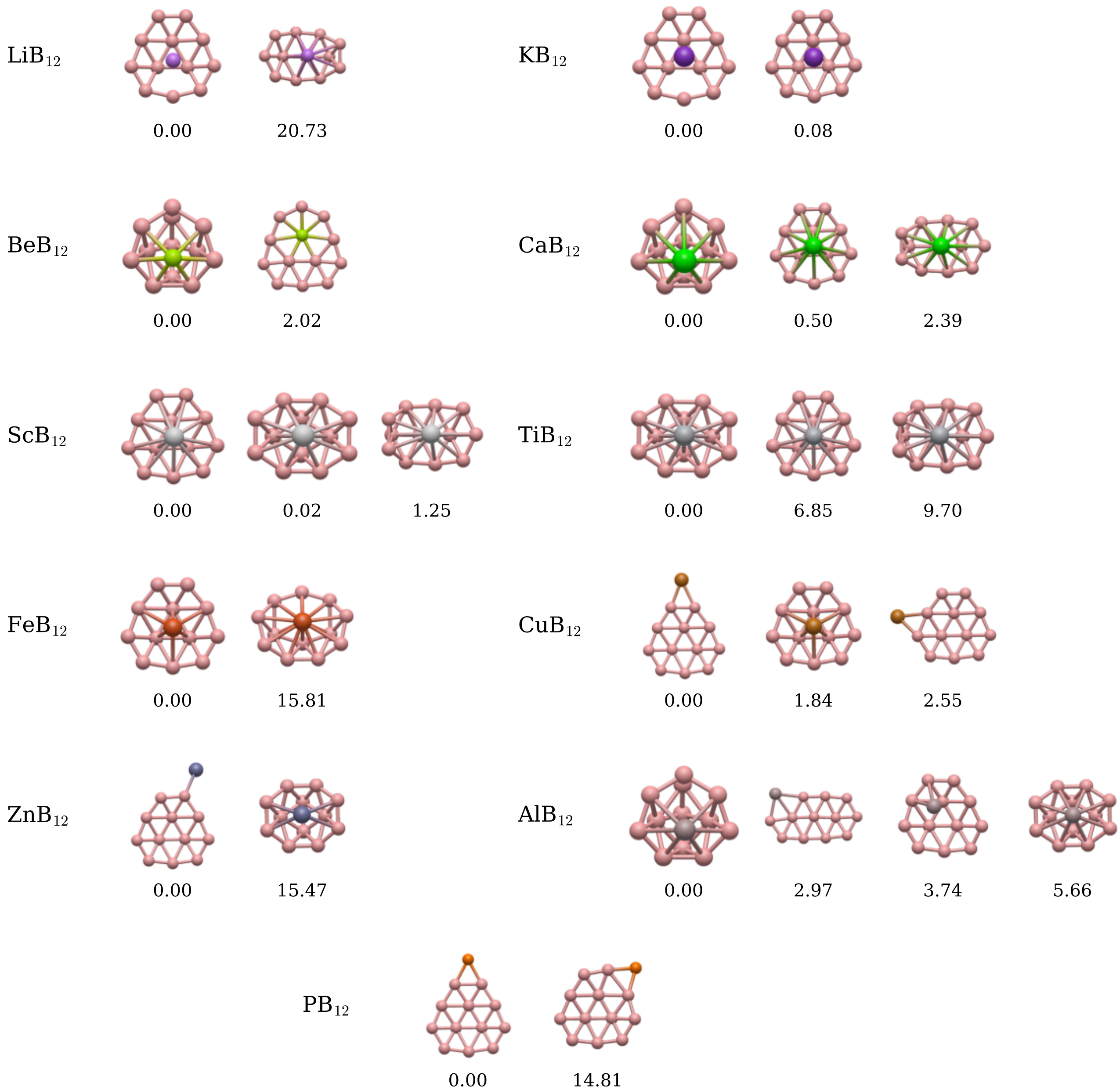

Figure S1: Representative low-lying isomers of the MB_{12} clusters considered in this work, with M = Li, K, Be, Ca, Sc, Ti, Fe, Cu, Zn, Al, and P. These structures were obtained from the global-minimum searches described in the main text at the PBE0/Def2-TZVP level of theory. Relative energies are reported with respect to the lowest-energy isomer of each composition. Cartesian coordinates and electronic energies for these structures are provided as separate `.xyz` files in **Section 1** of the Supporting Information.
